# The Pre-attentive L2 Orthographic Perception Mechanism Utilized by Bilinguals with Different Proficiency Levels

**DOI:** 10.3389/fpsyg.2017.01357

**Published:** 2017-08-07

**Authors:** Lijuan Liang, Michael Sharwood Smith, Vasiliki Chondrogianni, Baoguo Chen

**Affiliations:** ^1^Bilingual Cognition and Development Lab, School of English and Education, Guangdong University of Foreign Studies Guangzhou, China; ^2^Beijing Key Laboratory of Applied Experimental Psychology, School of Psychology, Beijing Normal University Beijing, China; ^3^Moray House School of Education, The University of Edinburgh Edinburgh, United Kingdom; ^4^School of Philosophy, Psychology and Language Sciences, The University of Edinburgh Edinburgh, United Kingdom

**Keywords:** Chinese–English bilinguals, L2 proficiency, pre-attentive, orthographic perception, orthographicsemantic connection

## Abstract

Language proficiency is predicted to modulate orthographic-semantic association in second language (L2) vocabulary acquisition, in accordance with the assumptions of the Developmental Bilingual Interactive-Activation model (BIA-d) (Grainger et al., 2010). The current study explored this modulation during pre-attentive L2 orthographic perception. ERPs were recorded from Chinese–English bilinguals with different L2 proficiency during their pre-attentive response to deviant and standard stimuli arranged in the oddball paradigm. Two stimulus types were investigated separately: L2 orthography and L1 orthography. In the L2 orthography condition, a MMN-N400 complex (i.e., deviancy effect) was found in the high L2 proficiency bilinguals, but only a marginally significant reduced negativity in an early time window was found in the low L2 proficiency bilinguals. In the L1 orthography condition, the high and low L2 proficiency bilinguals showed similar deviancy effect in the form of MMN-P3a-LPC complex. The current findings suggest that proficiency modulates pre-attentive L2 orthographic perception, such that the high L2 proficiency bilinguals activate the associated semantic representation instantly upon orthographic decoding, while the orthographic-semantic connection is not activated for the low L2 proficiency bilinguals. This is probably due to their difference in the strength of orthographic-semantic association. These findings contribute to the understanding of orthographic processing by bilinguals at the pre-attentive level and provide supporting evidence for the BIA-d model.

## Introduction

Most available information in the environment is pre-attentively (unconsciously) processed. Information after pre-attentive processing is ready to be selected for further attentive (conscious) processing (van der Heijden, 1996). The current study focused on orthographic processing of second language (L2) at the pre-attentive level, i.e., the unconscious, pre-attentive orthographic processing rather than conscious, overt orthographic recognition, since pre-attentive sensory processing may be a ready state which might “govern” some higher order attentive linguistic operations (Tiitinen et al., 1994). Ehri (2005) argues that the orthographic word recognition process seems to be unconscious and automatic. Readers are able to retrieve the pronunciations and meanings automatically upon the sight of the word form, with no need of the conscious attentional resources (LaBerge and Samuels, 1974; Guttentag and Haith, 1978; Pattamadilok et al., 2017).

### Orthographic Activation in Bilinguals

Orthographic processing in alphabetic languages minimally refers to the processing of the identities and positions of the constituent letters within a word (Grainger, 2008). Orthographic analysis in visual word recognition is the first ‘language-specific’ stage of the reading process (Grainger, 2008), and efficient orthographic processing guarantees the success of subsequent higher level language processing (Perfetti, 2010, 2011).

For bilinguals learning their L2 in formal instructional settings, the acquisition of new orthographic information is significant, because written language is substantially used in these learning contexts. Additionally, researchers have argued that L2 orthography has an important role for the shaping of linguistic representations (Veivo and Järvikivi, 2012; Veivo et al., 2016).

As for the cognitive processes implicated in orthographic activation, one popular model in this field, the Bilingual Interactive-Activation model (BIA+) by Dijkstra and van Heuven (2002), provides explanations for what happens during and after orthographic activation in bilinguals. In accordance with this model, a lexical representation has a resting-level activation determined by usage. For example, high frequency words are more easily and quickly activated than low frequency words. The resting level activation is associated with the orthographic-phonological-semantic nodes which form a highly interactive network. During word recognition, the sublexical orthographic representations will be activated by visual input, which leads to the activation of orthographic whole-word representations and sublexical phonological representations. Following this, the semantic representations as well as language membership representations (i.e., the language the word belongs to) will be activated by the orthographic and phonological whole-word representations (van Heuven and Dijkstra, 2010). Basically, after the activation of orthographic representations, linked phonological and semantic representations in the network become active as well. These assumptions have been supported by empirical evidence (Hauk et al., 2006).

Following the above theoretical assumptions, orthographic decoding will inevitably trigger the activation of any associated semantic information. However, it is also possible that if the orthographic-semantic association is weak, the orthographic-semantic connection might not be activated upon orthographic analysis. In this sense, the strength of orthographic-semantic connection was explored in light of the different L2 proficiency levels in the current study.

### Orthographic Perception as a Function of L2 Proficiency

Bilinguals can differ greatly in their proficiency in the second language despite similar learning environments (Roberts and Meyer, 2012; van Hell and Tanner, 2012). L2 proficiency, as a critical factor, is found to elicit certain linguistic-specific neural changes in a bilingual brain (Chee et al., 2001; Tatsuno and Sakai, 2005; Abutalebi and Green, 2007), and affect language processing in various aspects, such as orthographic processing (Veivo and Järvikivi, 2012; Veivo et al., 2016), bilingual lexical processing (van Hell and Tanner, 2012), morpho-syntactic processing (Osterhout et al., 2006; Steinhauer et al., 2009), and language switching (Costa and Santesteban, 2004; Garbin et al., 2011), etc. The current study aimed to further investigate the modulation of proficiency in orthographic processing.

Theoretically speaking, in accordance with the assumptions of the Developmental Bilingual Interactive-Activation model (BIA-d) (Grainger et al., 2010), the advanced version of the original BIA model that investigates second language vocabulary acquisition from a developmental perspective, proficiency modulates orthographic-semantic association. That is, more proficient learners get to the meaning from the orthographic stimulus faster than those that have a lower level of proficiency. In other words, the direct connection between the L2 word form representations and semantics is gradually strengthened with increasing L2 proficiency in bilinguals. These assumptions are consistent with the Revised Hierarchical model (RHM) (Kroll and Stewart, 1994), which presumes that as L2 speaker gains fluency, the indirect access to meaning (i.e., concepts) via L1 translation equivalents will gradually shift to direct connections from L2 word form to meaning.

Empirical evidence revealed the modulating effect of L2 proficiency in early orthographic processing (Veivo and Järvikivi, 2012). In this study, Finnish–French bilinguals were recruited and the masked cross-modal priming paradigm (67 ms SOA) was used to tap into the early stage of form processing by exploring the facilitation effect of within-language and between-language orthographic prime to L2 spoken word processing. Altogether, four prime types were included in the experiments: prime-target repetition (e.g., “stage-[staƷ]”), prime-target with orthographic overlap (e.g., “enne-ennui [ãnʯi]”), pseudo-homophones (e.g., “staje-[staƷ]”), and unrelated prime (e.g., “fueur-[staƷ]”). The results revealed more pronounced orthographic facilitation effect for prime-target repetition and prime-target with orthographic overlap in bilinguals with high L2 proficiency. A similar orthographic effect was not observed in bilinguals with lower intermediate proficiency. The authors concluded that proficiency modulates early orthographic and phonological processing in L2 spoken word recognition. In a follow-up study by Veivo et al. (2016) using the eye-tracking technique and the visual world paradigm, a facilitation effect of orthographic information was observed in a prime-target matching task in bilinguals with higher L2 proficiency in a time-window from 400 to 700 ms, whereas no such effect was found in those with lower L2 proficiency. Thus it was concluded that the activation of orthographic information in L2 spoken word recognition depends on L2 proficiency. Taken together, the above two studies provide evidence for the modulating role of L2 proficiency in orthographic perception, especially in the task of L2 spoken word recognition.

For L2 speakers learning the L2 through formal instruction, L2 orthographic processing is an integral part of their learning experience. This intensive experience with the written modality and its orthographic code may result in a strong orthographic-semantic connection as L2 proficiency increases. This may further result in a more automatic processing of L2 orthographic-semantic nodes that may be detected even at a pre-attentive stage. However, up until now, there is no evidence as to whether language proficiency modulates L2 orthographic perception at the pre-attentive level. The present study addresses this question by investigating two groups of bilinguals with different L2 proficiency who are learning their L2 (English) through formal instruction, where the written modality is predominantly used.

### Measuring Pre-attentive Orthographic Perception

The current study aimed to examine pre-attentive orthographic perception using event-related brain potentials (ERP) technique. Previous studies have often used offline (i.e., conscious processing) measures of orthographic processing, e.g., different letter string choice task, orthographic choice task, and homophone choice task, etc. (Cunningham et al., 2001; Deacon et al., 2009, 2012). While these tasks offer a comprehensive view of one’s orthographic knowledge and behavioral orthographic performance, they could not provide online measurement of ongoing brain responses of orthographic processing.

The research paradigms and techniques used to measure pre-attentive visual processing can also be used to explore pre-attentive orthographic processing, since orthographic information is a kind of linguistic-specific visual information. To be specific, some previous studies investigating pre-attentive visual processing used techniques like ERP which taps into different real-time cognitive processes with its greater temporal resolution and also reveals the neural basis of cognitive processing (Stefanics et al., 2015). Additionally, the oddball paradigm for the arrangement of standard and deviant stimuli was often used (e.g., Thierry et al., 2009).

One ERP component, visual mismatch negativity (vMMN), is often elicited by a visual deviancy with a peak latency falling in the range of 70–280 ms after stimulus onset at the parieto-occipital region or inferior temporal region (Pazo-Alvarez et al., 2003; Maekawa et al., 2005). It is a negative-going ERP component which represents pre-attentive change detection, requiring no active response on the part of the participants (Czigler et al., 2002, 2004; Winkler et al., 2005).

Another ERP component, a positive-going deflection termed P3a, sometimes follows the MMN component, indicating the involuntary engagement of attention to changes in the stimuli (Polich, 2007; Jakoby et al., 2011). P3a is often elicited within 250–280 ms after stimulus onset at the fronto-central region.

In addition, the N400 component (peaking within 250–500 ms with the largest effect over centro-parietal electrode sites) representing semantic analysis may also be elicited on orthographic processing, which is to be expected in the light of the assumed direct orthographic-semantic connection mentioned above. In other words, the associated semantic information is expected to become active as well after the activation of orthographic information, which is indicated by the presence of the N400 (Holcomb and Grainger, 2006, 2007). Therefore, it is possible that the N400 component will follow the MMN and P3a in processing orthographic anomaly, indicating the mapping of words forms onto semantic representations (Grainger and Holcomb, 2009).

Taken together, the ERP components of MMN, P3a, and N400 are expected to be elicited in the current experiment. Following the line of reasoning about orthographic-semantic connection, if orthographic decoding triggers the activation of semantic representation, an ERP pattern of MMN-P3a-N400 might be observed. If the orthographic-semantic connection is not activated, the N400 component may not be evoked and a pattern of MMN-P3a might be observed.

### The Current Study

In sum, we used the ERP technique to explore whether language proficiency modulates L2 orthographic perception at the pre-attentive stage of visual word recognition in Chinese (L1) – English (L2) bilinguals acquiring L2 through formal instruction where written language is substantially used. The high temporal resolution of the ERP technique and the oddball paradigm allow us to examine the pre-attentive brain responses of bilinguals to the discrimination of the deviant and standard contrast in two stimulus types: L2 orthography (e.g., “travle”-“travel”) and L1 orthography (e.g., 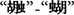). These two stimulus types were investigated in two separate blocks. L1 orthography was included as a control condition, so that we would observe whether the participants who were split into two groups according to their L2 proficiency level were similar to each other in processing L1 orthography in which they were equally highly experienced.

The deviant stimulus in the oddball paradigm differed from the standard stimulus in letter sequence which is a critical characteristic of orthographic representation, but not in constituent letters and visual complexity. To eliminate the interference of attention, a pre-attentive state was created in which participants were asked to listen to a story, press button when a red cross appeared on the screen, and ignore other visual stimuli. Five consecutive time windows (80–150 ms, 180–280 ms, 280–380 ms, 400–500 ms, and 500–600 ms) were analyzed in the ERP data, and the main deviancy effect (as reflected by the MMN, P3a, and N400 ERP components) is expected to be elicited in these time windows.

In accordance with the design of the current study, we expect to see a MMN (largest over the parieto-occipital region or inferior temporal region) — P3a (largest over the fronto-central region) — N400 (largest over the centro-parietal region) complex elicited by the deviant stimuli as compared to the standard stimuli in the L2 and L1 orthography conditions. If language proficiency does not affect L2 orthographic perception, we expect to observe similar ERP responses in bilinguals with high and low L2 proficiency for the two stimulus types. Otherwise, the low L2 proficiency bilinguals are expected to show different brain responses from the high in the L2 orthography condition, but probably not in the L1 orthography condition. Theoretically speaking, if bilinguals with high L2 proficiency show a deviancy effect in the form of the MMN and the N400 whereas those with low L2 proficiency show a different ERP pattern in which the N400 is not elicited, the prediction of the BIA-d model about the modulation of proficiency in the strength of orthographic-semantic connection would be supported.

## Materials and Methods

### Participants

The participants were 40 Chinese (L1) – English (L2) bilinguals, who are all college students from Beijing Normal University. They had begun receiving formal instruction of English at middle school and had not been to the English-speaking countries before. Written language is predominantly used in this L2 instruction setting. They were recruited based on five participant-selection criteria: duration of English language learning, College English Test-Band 6 (CET 6), Oxford placement test, self-rating of L1 skills, and self-rating of L2 skills. The CET 6, designed by the Ministry of Education of China, is used in all universities in China to evaluate the English proficiency of non-English majors. It consists of tasks on listening comprehension, reading comprehension, vocabulary knowledge, grammar knowledge and writing. The total score is 710, and the cutoff point (set by the Ministry of Education) for success and failure in the test is 427. The Oxford Placement test includes 25 multiple choice questions and a cloze test, and the total score is 50. The self-rating of L1 and L2 skills was based on the six-point scale assessment (1 for “quite poor,” 6 for “highly proficient”). These tests were proved to be valid measures of overall language proficiency (Hulstijn, 2012).

The participants were divided into two groups based on their overall L2 proficiency level, specifically their scores in the College English test, Oxford Placement test, and L2 self-rating test: 20 high L2 proficiency bilinguals (mean age = 22.4, *SD* = 2.0; 10 female), and 20 low L2 proficiency bilinguals (mean age = 22.8, *SD* = 2.6; 13 female). The duration of L2 learning, L2 proficiency, and L1 proficiency of both participant groups are presented in **Table 1**.

**Table 1:** Participant characteristics of the high and low L2 proficiency groups (SD).

	TS	CET	OPT6	L2- L	L2-R	L2-S	L2-W	L1-L	L1-R	L1-S	L1-W
High	10.6 (1.6)	568.9 (38.2)	41.5 (3.5)	4.5 (0.7)	3.6 (1)	4.1 (0.7)	3.8 (1.3)	5.1 (0.8)	4.8 (0.7)	4.6 (0.6)	4.7 (0.7)
Low	10.1 (1.4)	437.3 (34.6)	36.3 (4.3)	3.6 (1)	2.6 (1.3)	3.5 (0.8)	2.9 (1.4)	5.1 (1)	4.8 (1)	4.2 (0.8)	4.3 (1)

*TS, time spent on L2 learning (years); CET 6, College English Test-Band 6; OPT, Oxford Placement test; L, listening; R, reading; S, speaking; W, writing*.

The two groups were matched on age, duration of L2 learning, and the four L1 skills (all *p*s > 0.10). The overall L2 proficiency level of more proficient bilinguals was significantly higher than that of the less proficient bilinguals, according to their scores in the College English test, *t*(38) = 11.41, *p* < 0.001, Cohen’s *d* = 3.7, Oxford Placement test, *t*(38) = 4.08, *p* < 0.001, Cohen’s *d* = 1.33, and L2 self-rating test [listening: *t*(38) = 3.11, *p* < 0.005, Cohen’s *d* = 1.01; reading: *t*(38) = 2.57, *p* < 0.05, Cohen’s *d* = 0.83; speaking: *t*(38) = 2.15, *p* < 0.05, Cohen’s *d* = 0.7; writing: *t*(38) = 2.21, *p* < 0.05, Cohen’s *d* = 0.7].

All the participants were right-handed, had normal or corrected-to-normal vision, and had no history of hearing or language difficulties, neurological or psychiatric impairment based on self-report. Ethical approval was obtained from the Committee of Protection of Subjects at Beijing Normal University. They signed a consent form before the experiment, and were paid for their participation.

### Stimuli

In the oddball paradigm, the standard and deviant stimuli in each stimulus type differed only in sequence, but not in constituent letters and visual complexity.

In the L2 orthography condition, a familiar word “travel” with six letters and two syllables, which was learned in the first year of English learning during middle school and was used a lot in daily life, was chosen as the standard stimulus. The deviant stimulus “travle” was created by transposing two latter letters in the word, which disrupts the second syllable but would not change the pronunciation of the first syllable so that the magnitude of phonological priming effect could be matched as much as possible between the standard and deviant stimuli. Otherwise, the transposition of the first letters would make the deviant stimulus totally opaque in pronunciation. Besides, the phonotactic probability of the deviant stimulus “travle” was computed using the online calculator called “BLICK” developed by Bruce Hayes (Version 1.0, 2012). The score of “travle” is 9.965^1^, suggesting that this non-word is hardly pronounceable as a whole. On the whole, the transposition of the constituent letters in the present study is a valid measure of orthographic perception, according to the definition of orthography processing by Grainger (2008). In the L1 orthography condition, the character 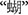 (hu1)^2^ was chosen because it consists of two parts, the semantic radical 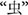 (chong2) and the phonetic radical 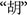 (hu1). Thus the transposition of the two parts in the deviant stimulus creates a pseudo-character 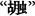 but would not incur changes to the original semantic and phonological cues. The materials used in the current study are presented in **Table 2**.

**Table 2:** Standard and deviant stimuli for the two stimulus types.

Stimulus type	Standard	Deviant
L2 orthography	**travel**	**travle**
L1 orthography	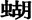	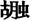

Altogether, the two stimulus types formed two blocks. In each block, the deviant stimulus were presented for 60 times (a probability of 16.66%), and the standard stimulus for 300 times (83.33%).

To eliminate the interference of attention, a pre-attentive state was created, in which the attention of the participants was attracted by listening to a story read in Chinese while they were watching the screen to detect the appearance of a red cross “+”. The story was new to all the participants, so they were easily attracted to it. Besides, the red cross detection task, in which the sign “+” was presented randomly for 40 times in each block, guaranteed that the participants watched the screen the whole time.

### Procedure

The order of the blocks was randomized across the participants. The standard and deviant stimuli in each block were presented on the screen in random order. Each stimulus was presented for 300 ms, and the inter-stimulus interval was 1000 ms. The red cross “+” appeared on the screen randomly among the trials in each block. The listening story was played during the whole experiment. Participants were asked to listen to the story attentively, press button when a red cross appeared on the screen, and ignore other visual stimuli. After the experiment, the participants were required to finish a listening comprehension test in which 10 multiple-choice questions were included to check whether they focused on the story or not. The total score of the test is 10. After this test, there was an informal interview with the participants asking them to translate orally five English words into Chinese: “drink,” “travel,” “watch,” “teach,” and “dance” (i.e., the word used for stimulus was mixed with four unrelated words to keep the participants blind as much as possible to the purpose of this study). The purpose of this interview was to see whether the participants knew the meaning of the stimulus to further check the material validity.

### Recording and Analysis

Electroencephalogram (EEG) was recorded at a 1000 Hz sampling rate using a 64-channel NeuroScan net. Eye movements were measured using vertical (electrodes below and above the left eye) and horizontal (two electrodes placed lateral to the outer canthi of the two eyes) electrooculogram. The common EEG and electrooculogram (EOG) reference was attached to the left mastoid, and re-referenced off-line to the mean of the activity at the left and right mastoids. Electrode impedances were kept below 5 kΩ. The electrophysiological signals were filtered on-line with a bandpass of 0.05–100 Hz and later low-pass (30 Hz) filtered off-line.

Electroencephalogram data analysis was performed using Scan 4.3. Eye movements were corrected by means of correlation. Epochs time-locked to the onset of the standard and deviant stimuli were extracted from -200 to 800 ms, and were averaged off-line for each group of stimuli separately for the two stimulus types for each participant. Baseline correction was performed in reference to pre-stimulus activity (-200 to 0 ms). Epochs with EEG exceeding either ±100 μV at any channel within intervals of 200 ms were automatically rejected off-line.

Five consecutive time windows were analyzed based on the visual inspection of the ERP data, with each time window centered on the peak of an ERP component elicited by the two participant groups: 80–150 ms, 180–280 ms, 280–380 ms, 400–500 ms, and 500–600 ms. Consecutive time windows, which were used a lot in the previous studies (Lavric et al., 2007, 2011), would provide fine temporal changes of the brain response after the onset of the stimulus.

In order to investigate the topographic distribution of the relevant effects, eleven ROIs were computed. ERP components were quantified using mean amplitude measures across the 11 ROIs. Data from midline and lateral electrodes were treated separately. Specifically, the midline electrode groups include: midline fronto-central (FZ, FCZ), midline centro-parietal (CZ, CPZ), and midline parieto-occipital (PZ, POZ). The lateral electrode groups include: left fronto-central (F1, F3, F5, FC1, FC3, FC5), left centro-parietal (C1, C3, C5, CP1, CP3, CP5), left parieto-occipital (P1, P3, P5, PO3, PO5, PO7), left temporal (T7, TP7), right fronto-central (F2, F4, F6, FC2, FC4, FC6), right centro-parietal (C2, C4, C6, CP2, CP4, CP6), right parieto-occipital (P2, P4, P6, PO4, PO6, PO8), right temporal (T8, TP8). The terms “fronto-central,” “centro-parietal,” and “parieto-occipital” used here correspond to “anterior,” “central,” and “posterior” used in some previous studies (Xu et al., 2013). Data for the L2 and L1 orthography conditions were analyzed separately, as L1 orthography was included only as a control condition. Repeated measures ANOVAs on the mean amplitudes over midline electrodes included deviancy (standard and deviant) and electrode region (fronto-central, centro-parietal, and parieto-occipital) as the within-subjects factors, and proficiency (high and low) as a between-subjects factor. Repeated measures ANOVAs over lateral electrodes included deviancy (standard and deviant), hemisphere (left and right), and electrode region (fronto-central, centro-parietal, parieto-occipital, and temporal) as the within-subjects factors, and proficiency (high and low) as a between-subjects factor. Since the main concern of the present study was the presence of deviancy effect in the L2 orthography condition as a function of L2 proficiency, only when reliable interactions involving deviancy and proficiency were found, further analysis was performed. Significance levels of the *F* ratios were adjusted with the Greenhouse–Geisser correction where appropriate and the corrected *p*-values are reported.

## Results

### Behavioral Results

In the listening comprehension test of the story, the score for the high L2 proficiency bilinguals was 9.55 (*SD* = 0.76), and for the low was 9.4 (*SD* = 0.82). There was no difference between the two participant groups, *t*(38) = 0.6, *p* = 0.55, Cohen’s *d* = 0.19. This means the attention of the participants was attracted by the listening story.

The detection accuracy of the red cross “+” was 98.5% (*SD* = 1.7%) for the high L2 proficiency bilinguals, and 98.7% (*SD* = 1.7%) for the low. There was no difference between the two participant groups, *t*(38) = -0.27, *p* = 0.78, Cohen’s *d* = -0.09. This means the participants watched the screen during the experiment.

Overall, the above behavioral results showed that the pre-attentive state was successfully created.

Also, all the participants had no difficulty at all in translating orally the five English words into Chinese in the informal interview after the experiment, suggesting that all the participants knew the meaning of the L2 stimulus, so its validity was guaranteed.

### ERP Results

**FIGURE 1:**
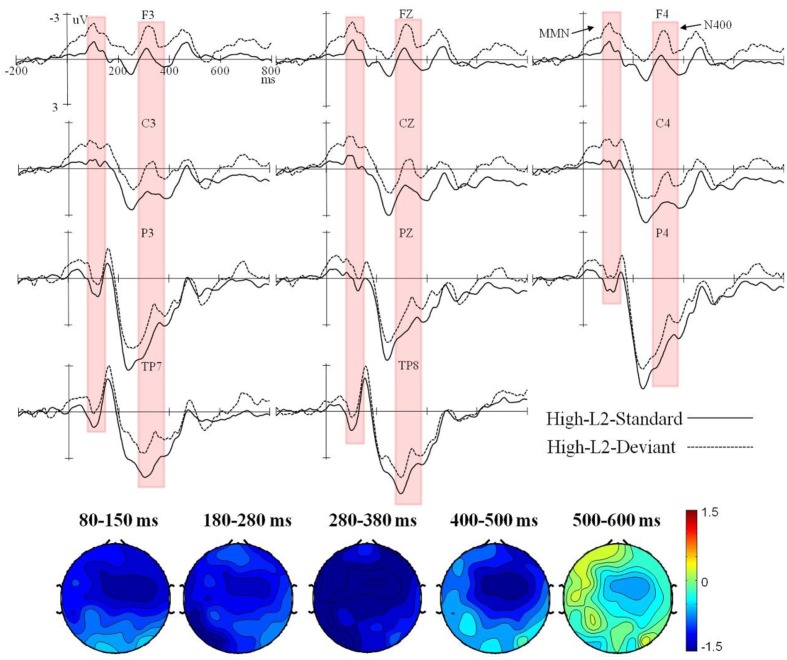
Grand average ERPs time-locked to the onset of the standard and deviant stimuli in the L2 orthography condition for the high L2 proficiency bilinguals, and the corresponding topographic maps for difference waves (deviant minus standard) within 80–150 ms, 180–280 ms, 280–380 ms, 400–500 ms, and 500–600 ms.

**FIGURE 2:**
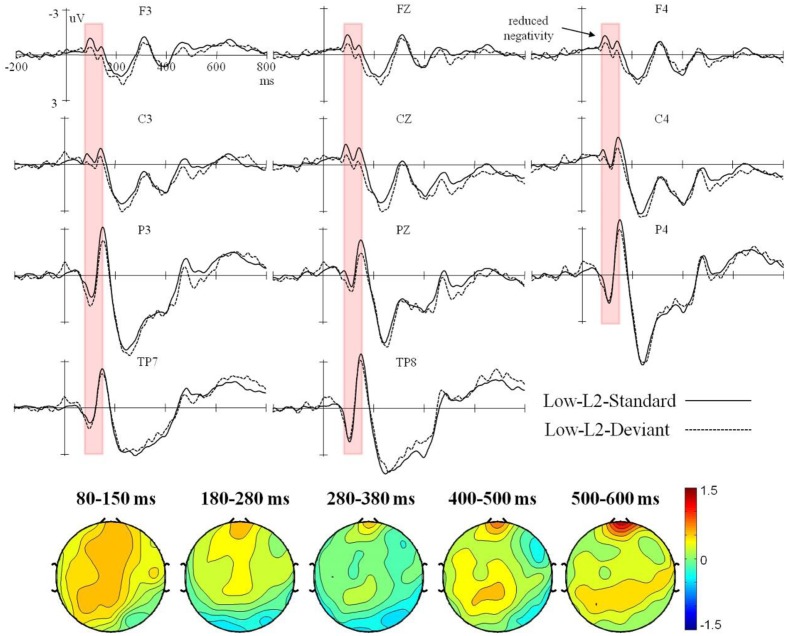
Grand average ERPs time-locked to the onset of the standard and deviant stimuli in the L2 orthography condition for the low L2 proficiency bilinguals, and the corresponding topographic maps for difference waves (deviant minus standard) within 80–150 ms, 180–280 ms, 280–380 ms, 400–500 ms, and 500–600 ms.

**FIGURE 3:**
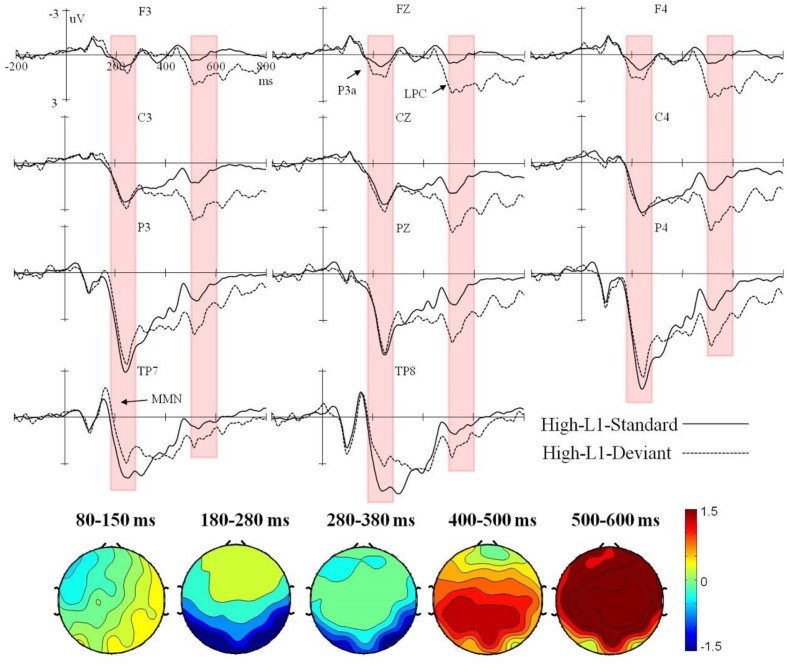
Grand average ERPs time-locked to the onset of the standard and deviant stimuli in the L1 orthography condition for the high L2 proficiency bilinguals, and the corresponding topographic maps for difference waves (deviant minus standard) within 80–150 ms, 180–280 ms, 280–380 ms, 400–500 ms, and 500–600 ms.

**FIGURE 4:**
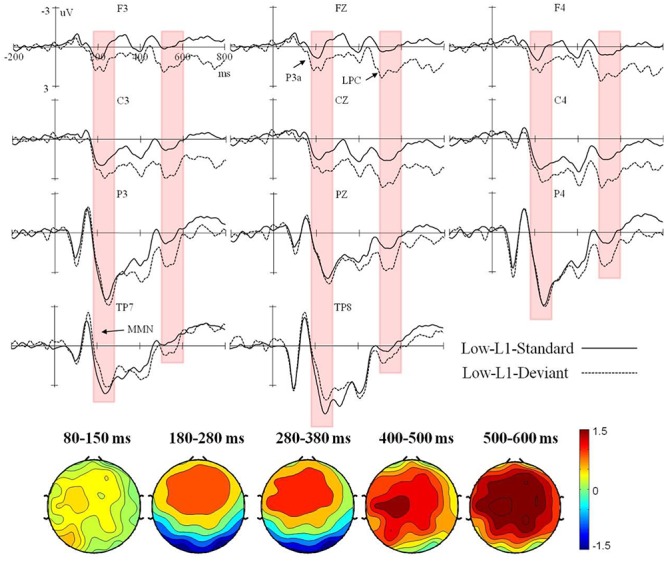
Grand average ERPs time-locked to the onset of the standard and deviant stimuli in the L1 orthography condition for the low L2 proficiency bilinguals, and the corresponding topographic maps for difference waves (deviant minus standard) within 80–150 ms, 180–280 ms, 280–380 ms, 400–500 ms, and 500–600 ms.

Event-related potential waveforms for the high and low L2 proficiency bilinguals in the L2 orthography and L1 orthography conditions, and the topographic maps (deviant minus standard) of the five consecutive time windows are presented in **Figures 1**–**4**.

#### 80–150 ms

In the L2 orthography condition, a significant interaction of deviancy × proficiency was found in both the lateral, *F*(1,38) = 9.98, *p* < 0.01, $\eta_{p}^{2}$ = 0.21, and the midline analyses, *F*(1,38) = 10.87, *p* < 0.01, $\eta_{p}^{2}$ = 0.22. Moreover, the four-way interaction of deviancy × hemisphere × electrode region × proficiency also reached significance in the lateral analysis, *F*(3,114) = 3.75, *p* < 0.05, $\eta_{p}^{2}$ = 0.09. Subsequent analysis by proficiency found a significant deviancy main effect in the high L2 proficiency bilinuals with the deviant stimuli eliciting a larger negative ERP response than the standard stimuli in both the lateral, *F*(1,19) = 8.65, *p* < 0.01, $\eta_{p}^{2}$ = 0.31, and the midline analyses, *F*(1,19) = 7.20, *p* < 0.05, $\eta_{p}^{2}$ = 0.27. However, an interaction of deviancy × hemisphere × electrode region, *F*(3,57) = 6.47, *p* < 0.01, $\eta_{p}^{2}$ = 0.25, was found in the low L2 proficiency bilinguals, and further analysis by electrode region found them showing only a marginally significant smaller negative ERP response for the deviant stimuli than the standard stimuli in the right fronto-central region, *F*(1,19) = 4.09, *p* = 0.057, $\eta_{p}^{2}$ = 0.18.

In the L1 orthography condition, only a significant interaction of deviancy × hemisphere × proficiency was found in the lateral analysis, *F*(1,38) = 4.83, *p* < 0.05, $\eta_{p}^{2}$ = 0.11. However, further analysis by proficiency did not reveal a significant effect of deviancy in either participant group (all *p*s > 0.05).

#### 180–280 ms

In the L2 orthography condition, a significant interaction of deviancy × proficiency was found in both the lateral, *F*(1,38) = 5.42, *p* < 0.05, $\eta_{p}^{2}$ = 0.12, and the midline analyses, *F*(1,38) = 6.15, *p* < 0.05, $\eta_{p}^{2}$ = 0.14. Subsequent analysis by proficiency was performed. The high L2 proficiency bilinuals showed a significant main effect of deviancy [lateral: *F*(1,19) = 8.54, *p* < 0.01, $\eta_{p}^{2}$ = 0.31; midline: *F*(1,19) = 6.72, *p* < 0.05, $\eta_{p}^{2}$ = 0.26], but the low L2 proficiency bilinguals showed no deviancy effect at all [lateral: *F*(1,19) = 0.05, *p* = 0.83, $\eta_{p}^{2}$ = 0.003; midline: *F*(1,19) = 0.67, *p* = 0.42, $\eta_{p}^{2}$ = 0.03].

In the L1 orthography condition, a significant interaction of deviancy × electrode region was found in both the lateral, *F*(3,114) = 40.96, *p* < 0.001, $\eta_{p}^{2}$ = 0.52, and the midline analyses, *F*(2,76) = 22.71, *p* < 0.001, $\eta_{p}^{2}$ = 0.37. Further lateral analysis by electrode region revealed a significant deviancy effect in the fronto-central, *F*(1,38) = 8.95, *p* < 0.01, $\eta_{p}^{2}$ = 0.19, and bilateral temporal regions, *F*(1,38) = 6.42, *p* < 0.05, $\eta_{p}^{2}$ = 0.14. Specifically, in both the high and the low L2 proficiency bilinguals, the deviant stimuli elicited a larger positive ERP response in the fronto-central region, but a larger negative ERP response in the bilateral temporal regions. Further midline analysis by electrode region showed a significant deviancy effect only in the fronto-central region, *F*(1,38) = 11.80, *p* < 0.01, $\eta_{p}^{2}$ = 0.24, with the deviant stimuli eliciting a larger positive ERP response than the standard stimuli in both participant groups.

#### 280–380 ms

In the L2 orthography condition, the interaction of deviancy × proficiency was significant in the lateral analysis, *F*(1,38) = 4.23, *p* < 0.05, $\eta_{p}^{2}$ = 0.10, and marginally significant in the midline analysis, *F*(1,38) = 4.07, *p* = 0.051, $\eta_{p}^{2}$ = 0.09. Further lateral and midline analysis by proficiency revealed a significant deviancy main effect in the high L2 proficiency bilinguals [lateral: *F*(1,19) = 8.74, *p* < 0.01, $\eta_{p}^{2}$ = 0.31; midline: *F*(1,19) = 5.71, *p* < 0.05, $\eta_{p}^{2}$ = 0.23], with the deviant stimuli eliciting a larger negative ERP response than the standard stimuli. However, no deviancy effect was found in the low L2 proficiency bilinguals [lateral: *F*(1,19) = 0.07, *p* = 0.79, $\eta_{p}^{2}$ = 0.004; midline: *F*(1,19) = 0.02, *p* = 0.88, $\eta_{p}^{2}$ = 0.001].

In the L1 orthography condition, a significant interaction of deviancy × electrode region was found in both the lateral, *F*(3,114) = 17.72, *p* < 0.001, $\eta_{p}^{2}$ = 0.32, and the midline analyses, *F*(2,76) = 6.87, *p* < 0.01, $\eta_{p}^{2}$ = 0.15. However, further lateral and midline analysis by electrode region did not find significant deviancy effect in either participant group (all *p*s > 0.05).

#### 400–500 ms

In the L2 orthography condition, the midline analysis did not reveal any significant effect involving deviancy (all *p*s > 0.05). The lateral analysis showed a significant interaction of deviancy × electrode region × proficiency, *F*(3,114) = 4.57, *p* < 0.05, $\eta_{p}^{2}$ = 0.11. However, further analysis by proficiency found no deviancy effect in either participant group (all *p*s > 0.05).

In the L1 orthography condition, a significant deviancy main effect was found in both the lateral, *F*(1,38) = 4.34, *p* < 0.05, $\eta_{p}^{2}$ = 0.10, and the midline analyses, *F*(1,38) = 5.79, *p* < 0.05, $\eta_{p}^{2}$ = 0.13, with the deviant stimuli eliciting smaller negativity slope than the standard stimuli in both participant groups.

#### 500–600 ms

In the L2 orthography condition, no deviancy effect was found in either participant group in the lateral and the midline analysis (all *p*s > 0.05).

In the L1 orthography condition, a significant main effect of deviancy was found [lateral: *F*(1,38) = 12.92, *p* < 0.01, $\eta_{p}^{2}$ = 0.25; midline: *F*(1,38) = 18.72, *p* < 0.001, $\eta_{p}^{2}$ = 0.33], with the deviant stimuli eliciting more positive ERP responses than the standard stimuli in both the high and the low L2 proficiency bilinguals.

#### A Summary of the Results

A summary of the presence of the deviancy effect for the five consecutive time windows of the two stimulus types in the two participant groups is presented in **Table 3**.

**Table 3:** A summary of the presence of the deviancy effect for the two stimulus types.

Proficiency		80–150 ms	180–280 ms	280–380 ms	400–500 ms	500–600 ms
L2 orthography	High	√ (enhanced negativity)	√ (reduced positivity)	√ (enhanced negativity)	×	×
	Low	√ (marginally significant reduced negativity)	×	×	×	×
L1 orthography	High	×	√ (enhanced positivity in the fronto-central region, but enhanced negativity in the bilateral temporal regions)	×	√ (reduced negativity)	√ (enhanced positivity)
	Low	×	√ (same as the High)	×	√ (same as the High)	√ (same as the High)

*√, the deviancy effect was elicited; ×, the deviancy effect was not observed*.

Generally speaking, in the L2 orthography condition, the deviancy effect in the form of enhanced negativity within 80–150 ms elicited in the high L2 proficiency bilinguals could be recognized as the MMN component. Furthermore, the enhanced negative peak falling in the range of 280–380 ms and being maximal over the central regions (i.e., fronto-central and centro-parietal brain regions) could be reckoned as the N400 component, according to Kutas and Federmeier (2011) who said the effects of semantic manipulations could be manifested through the N400 almost immediately from about 200 ms (and peak before 400 ms) when processing a critical word — written, spoken, or signed. Therefore, a MMN-N400 complex was found in the high L2 proficiency bilinguals, but only a marginally significant reduced negativity within 80–150 ms was found in the low. In the L1 orthography condition, the enhanced negativity in the bilateral temporal regions within 180–280 ms could be recognized as the MMN, and the enhanced positivity in the fronto-central region as the P3a. Taken together, in the L1 orthography condition, a deviancy effect as reflected by the MMN-P3a-late positive component (LPC) complex was found in both the high and the low L2 proficiency bilinguals.

## Discussion

The present study aimed to explore whether bilinguals with different L2 proficiency levels utilize distinct L2 orthographic perception mechanisms at the pre-attentive level. To address this question, we selected L2 learner groups who differed in terms of proficiency but who had received extensive formal L2 instruction, where the medium of instruction was primarily the written modality. We expected that strong orthographic-semantic association may have been established for bilinguals with high L2 proficiency and this may lead to higher automatization of the spread of activation from the visual to the semantic system during visual word recognition at the pre-attentive stage.

The results from the L2 orthography condition showed that different ERP responses were elicited between bilinguals with high and low L2 proficiency, suggesting that language proficiency does play a modulating role. This is consistent with the findings of Veivo and Järvikivi (2012) and Veivo et al. (2016)’s studies, in which the modulating role of proficiency in orthographic processing was found in the L2 spoken word recognition task at the attentive level. Meanwhile, no difference was found between the two groups in the L1 orthography condition. The significance of the current findings is elaborated below.

In the L2 orthography condition, bilinguals with high L2 proficiency were quite sensitive to the orthographic anomaly in the sequence of the constituent letters at the pre-attentive level (as reflected by the MMN), and further semantic analysis was directly triggered by mapping the deviant word form onto the semantic representation which is associated with the correct word form (as reflected by the N400). In other words, the moment more proficient bilinguals start orthographic decoding in L2, the node for instant semantic analysis is activated (as reflected by the MMN-N400 complex), as predicted by the BIA+ model (Dijkstra and van Heuven, 2002; van Heuven and Dijkstra, 2010). However, bilinguals with low L2 proficiency may be sensitive to the orthographic deviancy to a certain degree at the pre-attentive level, as reflected by an early marginally reduced negativity (i.e., an opposite ERP response to that of bilinguals with high L2 proficiency), but their orthographic-semantic connection was not activated at this sensory stage as reflected by the lack of the N400. Taken together, it could be concluded that language proficiency modulates L2 orthographic perception at the pre-attentive level, such that the high L2 proficiency bilinguals activate the associated semantic representation instantly upon orthographic decoding, while the orthographic-semantic connection is not activated for the low L2 proficiency bilinguals.

One reasonable explanation for the above finding is that bilinguals with high L2 proficiency could have acquired more accurate orthographic knowledge in the L2, which has led to well-established orthographic representations and strong orthographic-semantic association, and further leading to a high resting-level activation of the orthographic-semantic connection. In contrast, less proficient bilinguals don’t have well-established orthographic representations and strong orthographic-semantic association, so the resting-level activation of the orthographic-semantic connection is much lower. Therefore, the orthographic perception differences between bilinguals with high and low L2 proficiency could be modeled by setting different resting-level accessibility for orthographic-semantic association. This assumption is illustrated in the Modular Online Growth and Use of Language framework (MOGUL) framework (Truscott and Sharwood Smith, 2004; Sharwood Smith and Truscott, 2014). In explanations based on the MOGUL architecture, frequency of usage has to do with internal processing within one or more specific modules. According to the results of the current study, the bilinguals with high proficiency have built up the requisite resting level activation of associated representations across their visual and conceptual (semantic) systems, thus promoting their regular and rapid co-activation.

Furthermore, the finding of the current study, i.e., an observation of a modulating role of language proficiency in pre-attentive L2 orthographic perception, may be of use for further developing the BIA-d model of L2 vocabulary acquisition (Grainger et al., 2010). Evidence from this study supports the prediction of the BIA-d model in the sense that the strengthening of L2 orthographic-semantic connection may come along with the increasing L2 proficiency level. However, the current evidence is not sufficient to specify how this modulation of proficiency functions in shaping the orthographic-semantic connection. Grainger et al. (2010) said this shift or developmental change is like a “magic moment” in the evolution of L2 proficiency. In their exposition of the BIA-d model, they proposed a “clamping” mechanism to explain how this change happens. To be specific, at the initial phase of L2 acquisition, there is a “clamping” (i.e., co-activation) of the L2 word form representation, the equivalent L1 word form, the corresponding semantic representation, and the L2 language tag. Then gradually with increasing exposure to L2 word forms, there develops a kind of L2 autonomy where the connection between the L2 word form and semantic features strengthens with no need for the clamping of the equivalent L1 word form any more, and meanwhile inhibitory connection develops from the L2 language node to L1 word form representations. These assumptions about how this specific qualitative shift happens are beyond the scope of the current study which only tapped into whether a modulation of proficiency exists as predicted by the BIA-d model. Further exploration is still needed to provide sufficient evidence to the functioning of this modulation.

In accordance with the current findings, bilinguals with high L2 proficiency may benefit from an efficient and deep pre-attentive L2 orthographic-semantic analysis before subsequent cognitive processes take place. For them, words may be recognized even while attention is diverted away. This seems a good preparation for what happens during cognitive processes, a kind of ready state which guarantees efficient cognitive processing in visual word recognition. However, the bilinguals with low L2 proficiency level are not capable of that. For them, L2 orthographic analysis at the pre-attentive level could not activate the semantic representation, and thus they fail in pre-attentive semantic retrieval. The results of this study were obtained from the quite familiar word, so the current findings could be generalized to other similar stimuli with high familiarity.

Speaking of their native language, the two groups have equally well-established orthographic representations because of abundant exposure and immersion. The current evidence showed that both high and low L2 proficiency bilinguals were sensitive to L1 orthographic deviancy by exhibiting attention-related MMN and P3a components. The reason why the N400 component for semantic analysis was not elicited in either of the two participant groups is probably that a special compound word from the native language Chinese was used here. Specifically, the single Chinese character 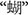 used in the current study does not form an independent semantic representation in the mental lexicon. Only when it is combined with 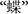, can the semantic activation for “butterfly” be triggered. Moreover, it is interesting to observe a LPC in the deviant stimulus in both groups. The LPC may reflect further analysis of the stimulus, and is sensitive to decision accuracy, perhaps confidence at the evaluation stage (Finnigan et al., 2002). Nevertheless, it could be concluded that bilinguals with high and low L2 proficiency use a similar L1 orthographic perception mechanism, since the two participant groups were same as each other in their brain responses.

Finally, one observation in the current study is that the latency of the MMN in the L1 orthography condition occurs later than in the L2 orthography condition (L2: within 80–150 ms, L1: within 180–280 ms). The reasons may be as follows: firstly, the L1 stimuli were not ideal since the Chinese stimulus was only half a compound with no semantic meaning by itself. However, it is very difficult to match L1 and L2 stimuli. In English, transposing the last two letters still makes the words readable, at least for the first half of the words. However, in Chinese, transposing the left and the right radicals could completely break Chinese orthography and make it unpronounceable. Secondly, this may be due to the different processing mechanisms of L1 and L2 orthography. According to Zhang et al. (2012), processing Chinese character relies on the extraction of two-dimensional form information at multiple levels including radicals, strokes, etc., which involves extensive and higher-level visual analysis implicating more visual resources. The study by Lin et al. (2011) and Su et al. (2012) found that a negativity at about 170 ms is associated with processing the orthography of Chinese characters. This negativity, which is specific for the very early identification of the orthography of Chinese characters occurs even later, at about 200 ms, in Zhang et al.’s (2012) study. However, processing alphabetic word only involves discrimination of one-dimensional linear combinations of letters or phonological units. In a word, dramatic contrast between the two types of orthography does exist, which leads to distinctive mental processes (Cao et al., 2015).

In sum, the current findings shed light on the distinct ways of pre-attentive L2 orthographic processing as a function of language proficiency by dividing participants into high proficiency and low proficiency groups. Further studies may treat proficiency as a continuous variable so as to explore the specific correlation between proficiency and online orthographic processing.

## Conclusion

Bilinguals with high L2 proficiency exhibited a MMN-N400 complex (i.e., deviancy effect) upon processing L2 orthographic anomaly at the pre-attentive level, while those with low L2 proficiency showed only a marginally significant reduced negativity in an early time window. These results contribute to the current literature, suggesting distinct ways of pre-attentive L2 orthographic processing between bilinguals with high and low L2 proficiency: the high L2 proficiency bilinguals activate the associated semantic representation instantly upon orthographic decoding, while the orthographic-semantic connection is not activated for the low L2 proficiency bilinguals. This is probably due to their difference in the strength of orthographic-semantic association. The current findings provide supporting evidence for the Developmental Bilingual Interactive Activation (BIA-d) model.

## Author Contributions

LL and BC designed the experiment and wrote the manuscript; LL collected and performed data analysis; MS, VC, BC edited and revised the manuscript.

## Conflict of Interest Statement

The authors declare that the research was conducted in the absence of any commercial or financial relationships that could be construed as a potential conflict of interest.

## Footnotes

1 The output numerical value of this calculator inversely reflects the phonotactic probability of the word, i.e., higher score indicating decreased phonotactic “goodness.” For example, the score for a perfect English word “ket” is zero, and for “nguhyee” is 12.295.

2 The number shows the character’s tone pronunciation in Mandarin.
